# Insights into Sexual Precocity of Female Oriental River Prawn *Macrobrachium nipponense* through Transcriptome Analysis

**DOI:** 10.1371/journal.pone.0157173

**Published:** 2016-06-09

**Authors:** Hongxia Jiang, Xilian Li, Yuhang Sun, Fujun Hou, Yufei Zhang, Fei Li, Zhimin Gu, Xiaolin Liu

**Affiliations:** 1 College of Animal Science and Technology, Northwest A&F University, Shaanxi Key Laboratory of Molecular Biology for Agriculture, Yangling, Shaanxi, People’s Republic of China; 2 Agriculture Ministry Key Laboratory of Healthy Freshwater Aquaculture, Key Laboratory of Freshwater Aquatic Animal Genetic and Breeding of Zhejiang province, Zhejiang Institute of Freshwater Fisheries, Huzhou, Zhejiang, People’s Republic of China; 3 College of Fisheries, Henan Normal University, Xinxiang, Henan, People’s Republic of China; Natural Resources Canada, CANADA

## Abstract

**Background:**

The oriental river prawn (*Macrobrachium nipponense*) is the most prevalent aquaculture species in China. The sexual precocity in this species has received considerable attention in recent years because more and more individuals matured at a small size, which devalues the commercial production. In this study, we developed deep-coverage transcriptomic sequencing data for the ovaries of sexually precocious and normal sexually mature *M*. *nipponense* using next-generation RNA sequencing technology and attempted to provide the first insight into the molecular regulatory mechanism of sexual precocity in this species.

**Results:**

A total of 63,336 unigenes were produced from the ovarian cDNA libraries of sexually precocious and normal sexually mature *M*. *nipponense* using Illumina HiSeq 2500 platform. Through BLASTX searches against the NR, STRING, Pfam, Swissprot and KEGG databases, 15,134 unigenes were annotated, accounting for 23.89% of the total unigenes. 5,195 and 3,227 matched unigenes were categorized by GO and COG analysis respectively. 15,908 unigenes were consequently mapped into 332 KEGG pathways, and many reproduction-related pathways and genes were identified. Moreover, 26,008 SSRs were identified from 18,133 unigenes. 80,529 and 80,516 SNPs were yielded from ovarian libraries of sexually precocious and normal sexually mature prawn, respectively, and 29,851 potential SNPs between these two groups were also predicted. After comparing the ovarian libraries of sexually precocious and normal sexually mature prawn, 549 differentially expressed genes (DEGs) and 9 key DEGs that may be related to sexual precocity of *M*. *nipponense* were identified. 20 DEGs were selected for validation by quantitative real-time PCR (QPCR) and 19 DEGs show consistent expression between QPCR and RNAseq-based differential expression analysis datasets.

**Conclusion:**

This is the first report on the large-scale RNA sequencing of ovaries of sexually precocious and normal sexually mature *M*. *nipponense*. The annotated transcriptome data will provide fundamental support for future research into the reproduction biology of *M*. *nipponense*. The large number of candidate SNPs and SSRs detected in this study could be used as genetic markers for population genetics and functional genomics in this species. More importantly, many DEGs, especially nine key DEGs between sexually precocious and normal sexually mature prawns were identified, which will dramatically improve understanding of molecular regulatory mechanism of sexual precocity of this species.

## Introduction

The oriental river prawn *Macrobrachium nipponense*, a member of the Palaemonidae family of decapod crustaceans, is a commercial freshwater prawn species that naturally distributed throughout most freshwaters and low-salinity estuarine regions in Japan, Korea, Vietnam, Myanmar, and China [1,2]. It is considered as an important fishery resource and widely farmed in China due to its flavor, high nutritive value and excellent adaptability, with an average annual production value of over 23 million USD [3]. However, sexual precocity has become prevalent with the expansion of production scale. Sexual precocity refers to early male and female gonad development, which has been observed in many crustaceans. Sexually precocious *M*. *nipponense* has a low value due to low growth rate, poor survival, and short life span [4–6], which seriously restricts the sustainable development of this species. The adverse effect of sexual precocity on female *M*. *nipponense* is particularly prominent. Sexually precocious female *M*. *nipponense*is far less than normal sexually mature female prawn in both weight and size. Some female prawns even become sexually mature at 0.5 grams, but fecundity is only 200–500 eggs. Eggs obtained from these females often result in poor-quality offspring. Thus, understanding the mechanisms that regulate ovarian maturation in female *M*. *nipponense* and controlling sexual precocity of this prawn is crucial to improving the production of this species.

The ovary is a multifunctional organ that plays a key role in reproduction and secretion of hormones for regulation of growth and development in female prawns [7]. Ovarian maturation in prawn is a complex process controlled by several factors, such as endocrine control, nutrition and environmental factors [8–11]. However, the molecular mechanisms involved in stimulating ovarian development in prawn are still unclear. Till now, some reproduction- and ovary development-related genes have been identified from ovaries in *M*. *nipponense*, such as *SUMO-conjugating enzyme* (*Ubc9*) gene [12], *heat shock protein 90* (*HSP90*) gene [13] and *Gonadotropin-releasing hormone receptor* (*GnRHR*) gene [14], etc. However, the genomic or transcriptomic level resources that come from *M*. *nipponense* ovary remain limited. So far, only one study has reported sequenced transcriptome from ovary of *M*. *nipponense*, which sequenced non-normalized EST library using traditional Sanger sequencing [15]. This recorded cDNA library contains 3256 ESTs that can greatly accelerate the research and discovery of new genes and molecular markers on *M*. *nipponense* ovary. However, the underlying mechanism of sexual precocity of this female prawn has not been fully revealed, especially at the molecular level, including genes and pathways. In a word, the lack of genomic and transcriptomic information of *M*. *nipponense* ovary poses an obstacle to identify genes and construct regulatory networks associated with sexual precocity of this prawn.

Recently, the development of next-generation sequencing (NGS) technologies such as Illumina HiSeq 2000 [16], ABI SOLiD and 454 of Roche [17], and the newly developed deep sequencing methods such as Solexa/Illumina RNA-seq and Digital gene expression (DGE) [18], have opened a new avenue into transcriptome characterization and gene-expression profiling for various species, and rapidly dominated transcriptome studies because the higher-accuracy, higher-speed and lower-cost than the first-generation sequencing technology (Sanger sequencing). The RNA-Seq, a technique based on sequencing the poly-A RNA fraction, is a powerful tool to study complex transcriptomes because it allows for not only characterizing isoforms from known genes but also discovering novel or predicted coding genes [19]. It gives a general view of gene expression especially in these species lack of a fully sequenced and assembled genome such as *M*. *nipponense*. Some cDNA libraries and transcriptome-level datasets have been obtained from *M*. *nipponense* by RNA-Seq to lay a foundation for functional genomics approaches used for improving the aquaculture performance of this species [2,20,21]. Based on these transcriptome studies, there are about 81,411 expressed sequence tags (ESTs) from *M*. *nipponense* in the public databases up to date. However, there have been no transcriptome studies regarding the ovary of sexually precocious *M*. *nipponense* was reported until now.

In the present study, we performed high-throughput sequencing of the ovaries of sexually precocious and normal sexually mature *M*. *nipponense* using Illumina RNA-Seq to generate a transcriptome database that will enlarge the public EST database for this species and help support future studies. The identification of differentially expressed genes and pathways in the ovary of these two types of prawn will help build a more complete understanding of the regulatory mechanisms associated with sexual precocity. In addition, the simple sequence repeats (SSRs) and single nucleotide polymorphisms (SNPs) reported in this transcriptome study are also potentially useful for population genetics and functional genomics studies in this species.

## Materials and Methods

### Sample preparation and RNA extraction

There were two groups of female experimental prawn, one group was sexually precocious *M*. *nipponense* (MNOP) (2.5–3.5 cm, 0.5–1.5 g) which has grown about 90 days from hatching to sexual maturity, another group was normal sexually mature *M*. *nipponense* (MNON) (4.5–5.5 cm, 2.5–4.5 g) which took about one year to reach sexual maturity after hatching. Each group had ten samples which were all obtained from aquaculture base in Zhejiang institute of freshwater fisheries, Huzhou, China, and their ovaries were all at the stage V (maturation stage) based on the external morphology, color, gonado-somatic index (GSI), and histological features [13,15]. After prawns were anesthetized and finally sacrificed by placing them in an ice bath, ovaries were dissected and snap frozen in liquid nitrogen and stored at– 80°C until use. The study was approved by the Institutional Animal Care and Use Ethics Committee of Agriculture Ministry Key Laboratory of Healthy Freshwater Aquaculture, Zhejiang Institute of Freshwater Fisheries (Huzhou, China). Total RNA was extracted using Trizol Reagent (Invitrogen, Carlsbard, CA, USA). The integrity and purity of the total RNA were detected by an Agilent-2100 bioanalyzer (Agilent Technologies, Inc., Santa Clara CA, USA) and its concentration was determined using the NanoDrop 2000 spectrophotometer (Nano-Drop, Thermo Scientific, Wilmington, DE, USA). Ten RNA samples from each group were pooled in equal amounts, generating two mixed RNA samples from MNOP and MNON respectively, which were then used to prepare two separate RNA-seq transcriptome libraries.

### Transcriptome library preparation and Illumina sequencing

RNA purification, reverse transcription, library construction and sequencing were performed at Shanghai Majorbio Bio-pharm Biotechnology Co., Ltd. (Shanghai, China) according to the manufacturer’s instructions (Illumina, San Diego, CA). The two RNA-seq transcriptome libraries were prepared using Illumina TruSeqTM RNA sample preparation Kit (San Diego, CA). Poly(A) mRNA was purified from total RNA using oligo-dT-attached magnetic beads and then fragmented by fragmentation buffer. Taking these short fragments as templates, double-stranded cDNA was synthesized using a SuperScript double-stranded cDNA synthesis kit (Invitrogen, CA) with random hexamer primers (Illumina). Then the synthesized cDNA was subjected to end-repair, phosphorylation and ‘A’ base addition according to Illumina’s library construction protocol. Libraries were size selected for cDNA target fragments of 200–300 bp on 2% Low Range Ultra Agarose followed by PCR amplified using Phusion DNA polymerase (New England Biolabs, Boston, MA) for 15 PCR cycles. After quantified by TBS380, two RNA-seq libraries were sequenced in single lane on an Illumina Hiseq 2500 sequencer (Illumina, San Diego, CA) for 2×100bp paired-end reads [22,23].

### Sequencing data assembly and functional annotation

Image data output from the sequencer was transformed into raw reads by base calling, and stored in FASTQ format. The raw reads were filtered into the high quality clean reads after removing adaptor sequences, empty reads, and low quality sequences (reads with unknown sequences ‘N’ or less than 20bp) using SeqPrep (https://github.com/jstjohn/SeqPrep)and Sickle(https://github.com/najoshi/sickle). The clean reads were then assembled *de novo* into transcripts using the Trinity program (http://trinityrnaseq.sourceforge.net/, version number: trinityrnaseq-r2013-02-25) [24]. To eliminate redundant sequences, transcripts were clustered based on sequence similarities and the longest transcript in each cluster represented the final unique sequence (unigene). All the unigenes were searched against the protein databases such as non-redundant (NR) protein sequence database, Kyoto Encyclopedia of Genes and Genomes (KEGG) database, Search Tool for the Retrieval of Interacting Genes (String) database, Swissprot and Pfam databases to identify the proteins with the highly sequence similarity to the given unigenes and retrieve their function annotations, using BLASTX with a typical cut-off *E*-value <1e^-5^. Biological processes, molecular functions and cellular components were described by GO annotations of unigenes using BLAST2GO (http://www.blast2go.com/b2ghome) [25]. Clusters of Orthologous Groups (COG) (http://www.ncbi.nlm.nih.gov/COG/) and KEGG (http://www.genome.jp/kegg/) were used to predict possible functional classifications and metabolic pathways, respectively [26,27].

### Molecular Marker Detection

MSATCOMMANDER V. 0.8.2 (http://code.google.com/p/msatcommander/) [28] was used to search for microsatellites (simple sequence repeats, SSRs). The minimum repeat lengths considered were as follows: ten repeats for mono-SSRs; six repeats for di-SSRs; and four repeats for tri-, tetra-, penta-, and hexa-SSRs. SSR-containing unigenes were annotated based on BLAST similarity searches. The location of SSRs was estimated based on open reading frames (ORFs) and untranslated regions (UTRs) within the unigenes that were identified using Trinity [24]. All the consensus assembly sequences generated from the two transcriptome libraries were employed as reference sequences to detect potential single nucleotide polymorphisms (SNPs) in MNON and MNOP using Samtools V. 0.1.18(http://samtools.sourceforge.net) [29] and VarScan v.2.2.7(http://varscan.sourceforge.net/) [30]. In addition, to identify SNPs between MNON and MNOP groups, all sequences generated from the MNON transcriptome library were employed as reference sequences and SNPs in MNOP were detected using Samtools V. 0.1.18 and VarScan v.2.2.7.

### Identification and annotation of differentially expressed genes

We used the RNASeq by Expectation Maximization (RSEM) (http://deweylab.biostat.wisc.edu/rsem/) [31] to calculate unigene abundances from RNA-Seq data. FPKM (fragments per kilobase of exon model per million mapped reads) was used to quantify the gene expression levels. FPKM value can be directly used for the comparison of gene expression level between samples. FPKM is calculated as follows:

$$FPKM=\frac{total\;exon\;Fragments}{mapped\;reads\;(Millions)\;\times exon\;length\;(kb)}$$

In this formula, total exon Fragments is the number of reads that aligned to a specific unigene, mapped reads (Millions) is the total number of reads that aligned to all unigenes, exon Length (kb) is the length of the unigene.

The detection of differentially expressed genes (DEGs) between ovaries of MNON and MNOP was performed using edgeR package (Empirical analysis of Digital Gene Expression in R) (http://www.bioconductor.org/packages/2.12/bioc/html/edgeR.html) [32] with the rigorous algorithm method. Following the procedures described in the edgeR documentation, read count tables were loaded into R, normalized using the default method for edgeR (trimmed mean of M values, or TMM), and then tested for differential expression using the Fisher exact Test method. 0.1, a typical value for the common biological coefficient of variation (square root-dispersion) for datasets arising from well-controlled experiments, was chosen as a nominal dispersion value for the data without biological replicates. The *P*-values reported by edgeR were adjusted for multiple hypothesis testing using the False Discovery Rate (FDR) control method [33], and the ratio of FPKMs was converted to the fold-change in the expression of each gene in two samples simultaneously. FDR < 0.05 and the absolute value of logFC (Fold change) ≥ 1 were set as the threshold for significantly differential expression. The function of DEGs was annotated by the GO and KEGG pathway enrichment analyses at a *P* ≤ 0.05.

### Quantitative real-time PCR (QPCR) validation

Twenty candidate DEGs (Thirteen annotated DEGs and seven unannotated DEGs) were randomly selected for validation by QPCR. Total RNA was extracted from ovaries of MNON and MNOP using Trizol Reagent (Invitrogen, Carlsbard, CA, USA), and the RNA samples were reverse-transcribed using PrimeScript^®^RT reagent Kit with gDNA Eraser (Perfect Real Time) (TaKaRa, Dalian, China). Oligonucleotide primers (S1 Table) were designed with the Primer Premier 5.0 software and synthesized by Invitrogen (Shanghai, China). QPCR was performed using SYBR^®^ Premix Ex Taq™ Ⅱ (Tli RNaseH Plus) (TaKaRa, Dalian, China) on a CFX96^TM^ Real-Time PCR Detection System (Bio-Rad, USA). Each PCR reaction (25 μL) contained 12.5 μL SYBR^®^ Premix Ex Taq Ⅱ, 0.4 μM of each primer and appropriately diluted cDNA. The thermal cycler program was 95°C for 30 s, followed by 40 cycles of 95°C for 5 s and 60℃ for 30 s. Six biological and three technical replicates were performed respectively. Beta-actin (*β*-actin) was amplified in parallel as an internal control for normalization of QPCR data due to its stable expression in *M*. *nipponense* after the expressions of three reference genes including *β*-actin, elongation factor 1-alpha (Ef1α) and ribosomal protein L8 (RpL8) were compared in different tissues of MNOP and MNON by the method of Qian *et al* (2014) [34]. The relative changes in gene expression levels were calculated using the 2^−ΔΔCT^ method [35,36]. Statistical analyses were performed with SPSS 20.0 software (SPSS Inc., Chicago, IL, USA). Results were presented as the means±S.D. (n = 6) for each group. One-way analysis of variance (ANOVA) followed by the post hoc test were carried out to determine whether the differences between groups were significant (*P* < 0.05).

## Results

### Transcriptome sequencing and assembly

**Fig 1:**
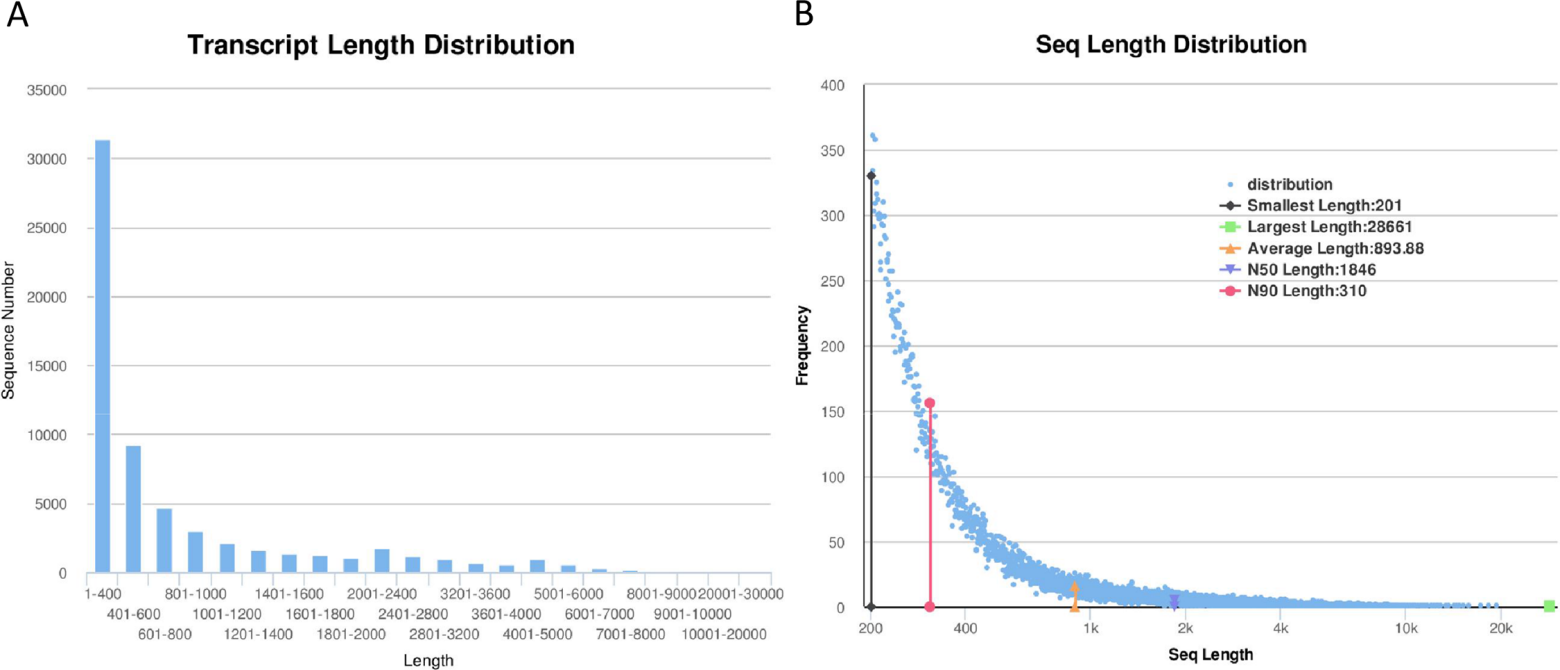
Length distribution of 63,336 assembled unigenes. X-axis: Size distribution of assembled unigenes, Y-axis: The number of unigenes in different length range.

Two cDNA libraries were constructed from ovaries of MNOP and MNON. The Illumina Hiseq 2500 sequencing generated 47,254,012 raw reads from the MNOP and 56,793,358 raw reads from the MNON. The raw sequencing data were deposited in the Short Read Archive (SRA) of the NCBI with accession numbers of SRP063589. After filtering out the adaptor primers and empty and low-quality reads, 42,115,012 and 50,789,578 clean reads were generated from MNOP and MNON libraries, respectively (Table 1). After *de novo* assembly of clean reads using Trinity, a total 63,336 unigenes were identified. The lengths of all unigenes were distributed as 893.88 bp, 28,661 bp, 201 bp and 1846 bp from average, longest, shortest and N50 length, respectively (Fig 1A). Of these, 31,441 (49.64%) were 1–400 bp; 9,273 (14.64%) were 401–600 bp; 4,634 (7.32%) were 601–800 bp; 3,021 (4.77%) were 801–1000 bp and 2,187(3.45%) were 1001–1200 bp (Fig 1B).

**Table 1:** Statistics of the sequencing results from two samples.

The sample name	Number of reads	Number of bases (bp)	Error%	Q20%	Q30%	GC%
Raw Data						
MNOP	47,254,012	4,772,655,212	0.0532	90.91	80.99	41.01
MNON	56,793,358	5,736,129,158	0.0503	91.41	81.87	41.04
Clean Data						
MNOP	42,115,012	4,036,709,557	0.0296	97.74	89.37	40.65
MNON	50,789,578	4,886,896,178	0.0288	97.86	89.85	40.71

### Functional annotation and classifictiaon

**Fig 2:**
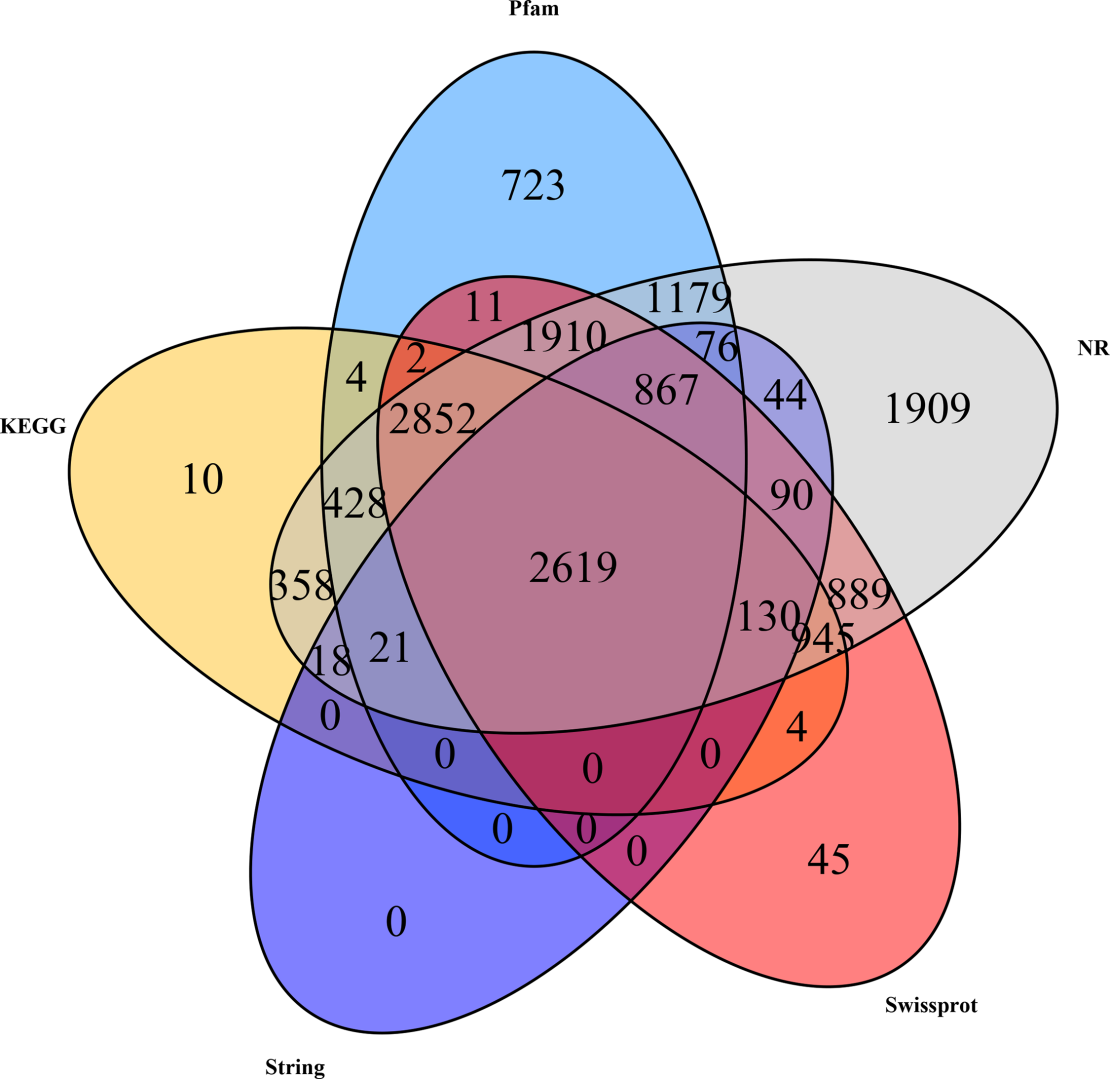
The Venn diagram shows the distribution of unigenes in different databases. The number of the shared unigenes are in the cross area, while the number of the specific unigenes are in the single area.

**Fig 3:**
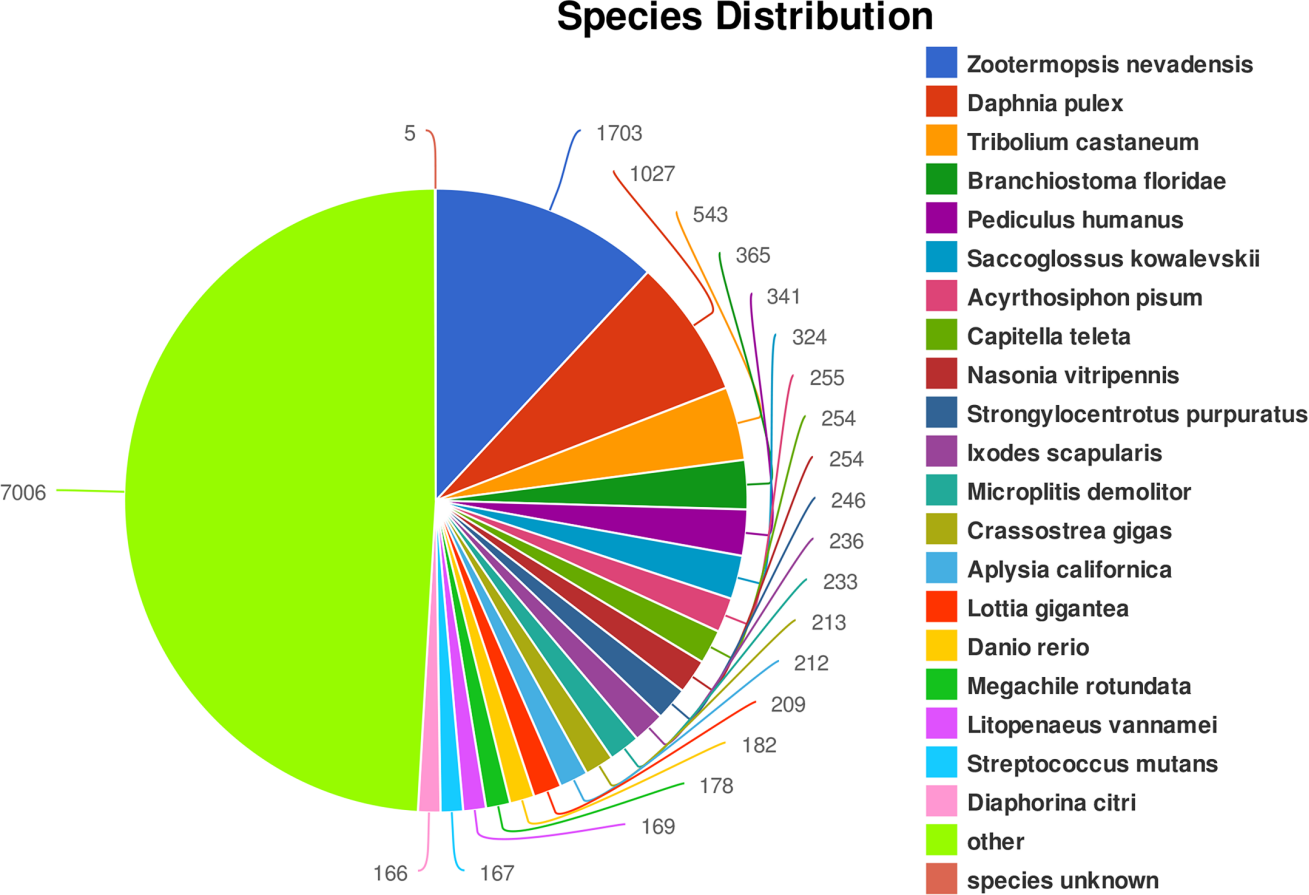
The top twenty species that match to the sequences of *M*. *nipponense*.

All the 63,336 assembled unigenes were searched against the public databases including NR, KEGG, String, Swissprot and Pfam databases, and a total of 15,134 unigenes were annotated, accounting for 23.89%. Unigenes that were annotated as unique in public databases are as follows: 10,692 unigenes in the Pfam database, 7,391 unigenes in the KEGG database, 3,865 unigenes in the String database, 10,364 unigenes in the Swissprot database, and 14,335 unigenes in the NR database. Besides, about 76.11% of unigenes (48,202) did not show any matches to known genes (Fig 2, Table 2). Of 14,335 unigenes that had BLAST matches against the NR database, 1703 matched unigenes (11.88%) showed significant homology with gene sequences from *Zootermopsis nevadensis*, followed by *Daphnia pulex* (1027, 7.16%), *Tribolium castaneum* (543, 3.79%), *Branchiostoma floridae* (365, 2.55%), *Pediculus humanus corporis* (341, 2.38%), and *Saccoglossus kowalevskii* (324, 2.26%). *Litopenaeus vannamei* (169, 1.18%) was the only decapod crustacean of the suborder Natantia in the top twenty species that match to the sequences of *M*. *nipponense* (Fig 3).

**Table 2:** Summary of the annotation percentage of *M*. *nipponense* as compared to public database.

Database	Number of unigenes	Annotation percentage (%)
Pfam	10,692	16.88%
KEGG	7,391	11.67%
String	3,865	6.102%
Swissprot	10,364	16.36%
NR	14,335	22.63%
All annotated unigenes	15,134	23.89%
Total unigenes	63,336	

**Fig 4:**
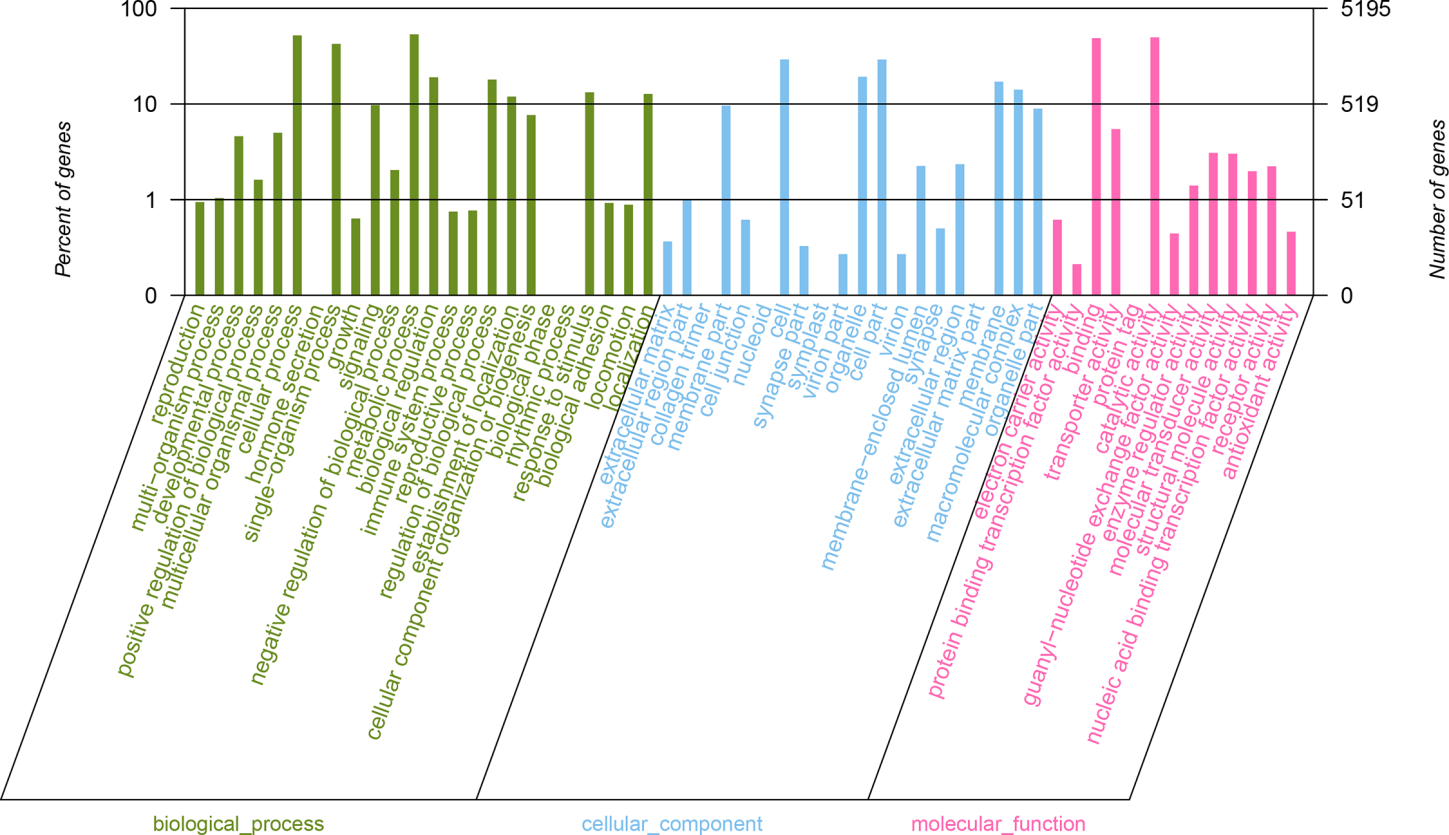
Gene ontology (GO) category of the unigenes. 5,195 annotated unigenes were classified into 3 functional categories: molecular_function, biological_process and cellular_component.

**Fig 5:**
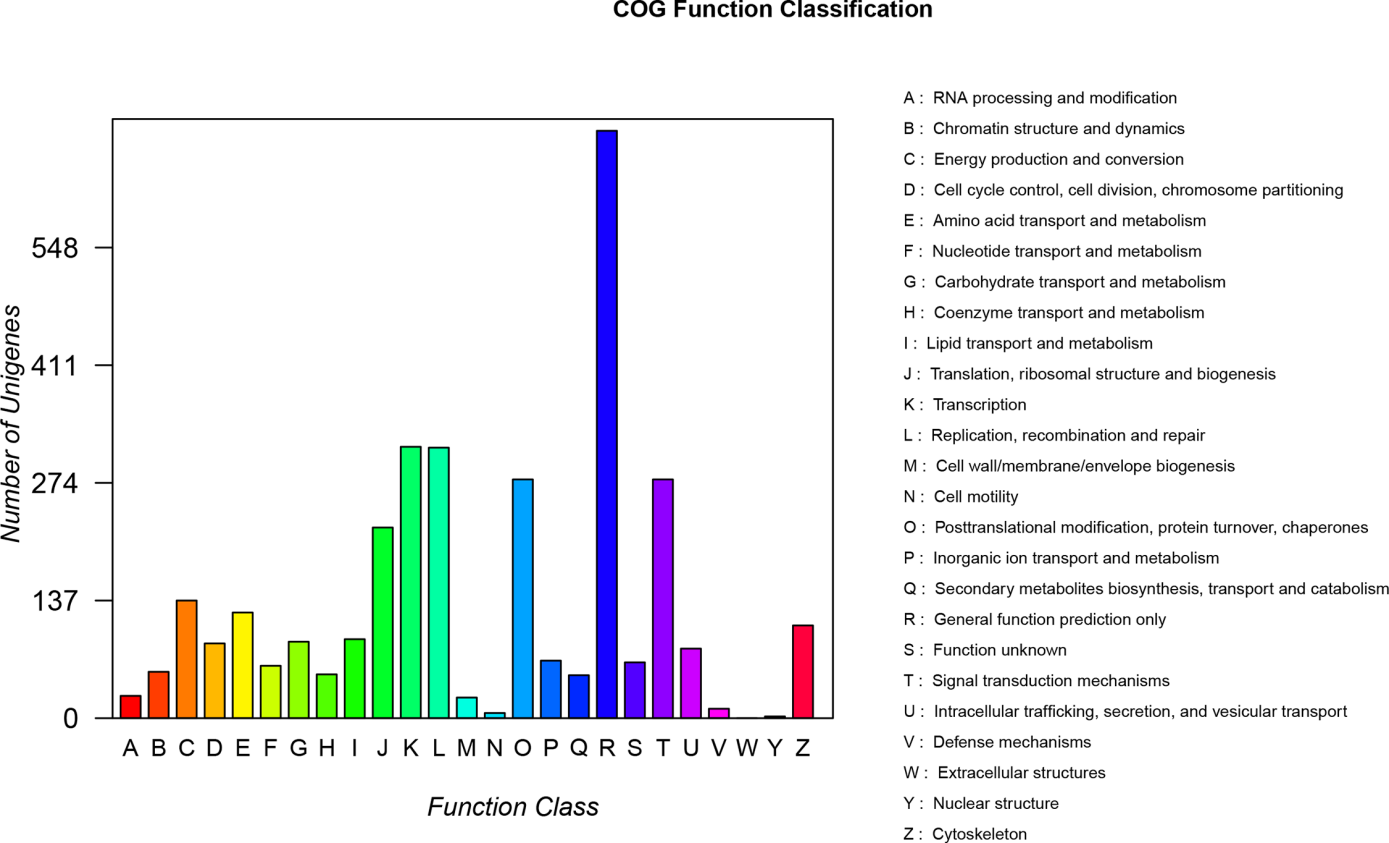
COG function classification of the unigenes. 3,227 unigenes were classified into 25 COG categories.

**Fig 6:**
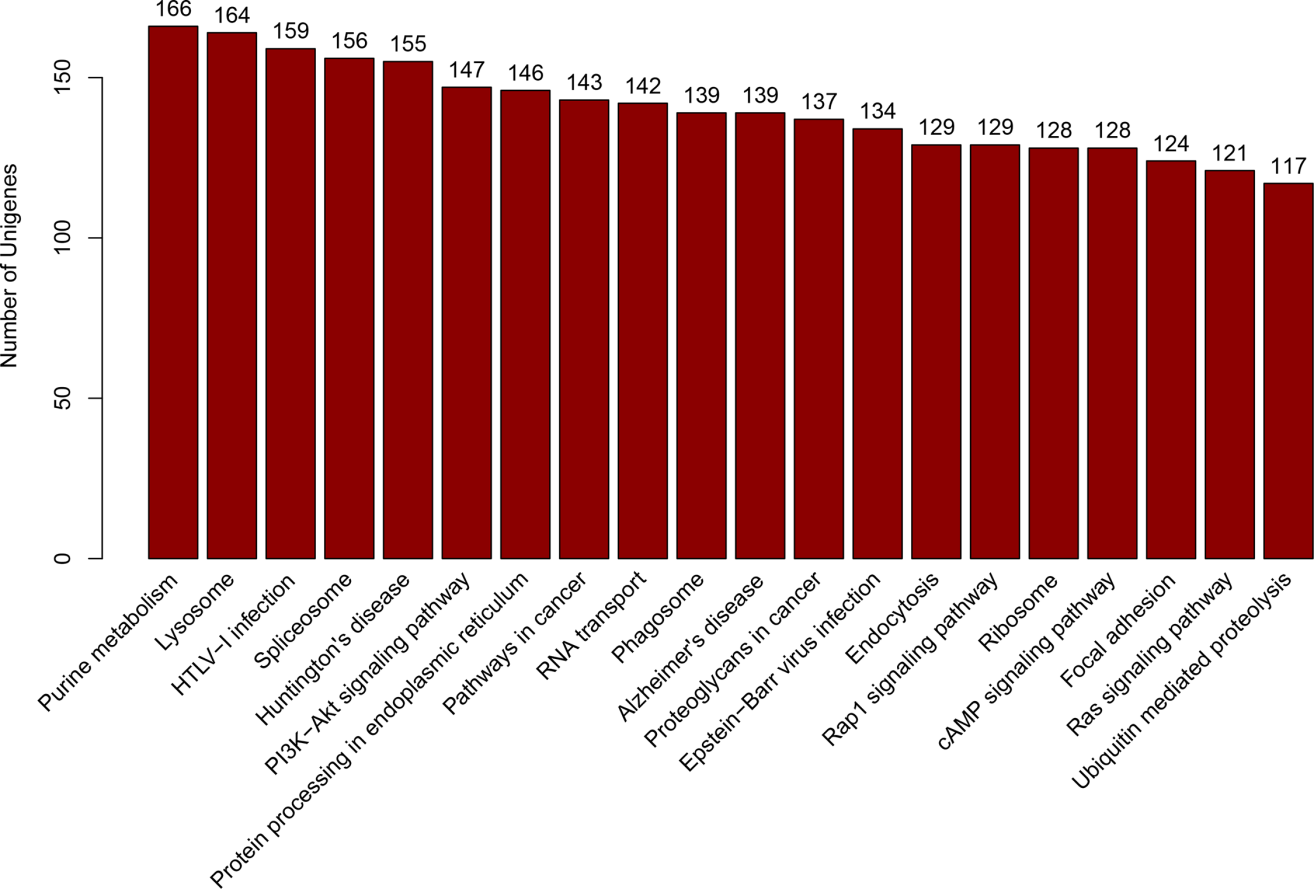
The top twenty most abundant KEGG pathways. X-axis: distribution of KEGG pathways, Y-axis: the number of sequences mapped into each KEGG pathway.

Gene Ontology (GO) and Cluster of Orthologous Groups of proteins (COG) analyses were used to classify the functions of unigenes. A total of 5,195 annotated unigenes were classified into 3 functional categories (level 2 terms): molecular function, cellular component and biological process (Fig 4). In the category of molecular function, unigenes were clustered into 13 classifications. The largest subcategory of the molecular function was ‘catalytic activity’ (2585, 49.76%) and the second was ‘binding’ (2536, 48.82%). In the category of cellular components, unigenes were divided into 20 classifications: the most represented cellular components were “cell” (1517, 29.20%), and ‘cell part’ (1516, 29.18%). In the category of biological processes, unigenes were grouped into 24 classifications: the most represented biological processes were ‘metabolic process’ (2780, 53.52%) and ‘cellular process’ (2710, 52.17%) (S2 Table). In addition, a total of 3,227 unigenes were clustered into 25 categories of COG (Fig 5). The largest category was ‘general function prediction only’ (684, 20.08%), the second category was ‘Transcription’ (316, 9.79%), and the third category was ‘Replication, recombination and repair’ (315, 9.76%) (S3 Table). To systematically analyze the active biochemical pathways in ovary of *M*. *nipponense*, unigenes were compared against the KEGG database using BLASTX and the corresponding pathways were established. A total of 7,391 unigenes were assigned to 332 KEGG pathways. ‘Purine metabolism’ pathway had the largest number of unigenes (166), followed by ‘Lysosome’ (164 unigenes), ‘HTLV-I infection’ (159 unigenes), ‘Spliceosome’ (156 unigenes), ‘Huntington's disease’ (155 unigenes) and ‘PI3K-Akt signaling pathway’ (147 unigenes) (Fig 6).

### Identification of reproduction-related pathways and genes

Among 332 KEGG pathways, some reproduction-related pathways were found, such as ‘Ovarian steroidogenesis’, ‘Progesterone-mediated oocyte maturation’, ‘MAPK signaling pathway’, ‘Wnt signaling pathway’, ‘Oocyte meiosis’, ‘GnRH signaling pathway’, ‘Estrogen signaling pathway’, ‘p53 signaling pathway’, ‘Steroid hormone biosynthesis’ and ‘Fatty acid biosynthesis’. As might be expected, a lot of reproduction-related genes were indentified from these pathways, for example, mitogen-activated protein kinase gene family (*MAPKs*), cell cycle dependent protein kinase gene family (*CDKs*), Wnt protein gene family (*WNTs*), adenylate cyclase gene family (*ADCYs*), anaphase-promoting complex gene family (*APCs*), heatshock protein 90 (*HSP90*), insulin-like receptor (*INSR*), insulin-like growth factor 1 receptor (*IGF1R*), fatty acid synthase (*FASN*), cyclooxygenase (*COX2*), protein kinase A (*PKA*), membrane-associated tyrosine- and threonine-specific cdc2-inhibitory kinase-like protein (*PKMYT*), cytoplasmic polyadenylation element-binding protein (*ORB*), p38 MAP kinase (*P38*) and low-density lipoprotein receptor-related protein 5/6 (*LRP5_6*) genes, *etc* (S4 Table). In addition, some other reproduction-related genes were also indentified from our KEGG database (S5 Table) based on comparisons with published data for crustaceans, for instances, cathepsin L **(***CTSL*), ribosomal protein L24 (*RPL24*), Broad-complex (*BR-C*), valosin-containing protein (*VCP*), protein disulfide isomerase A6 (*PDIA6*), adipose differentiation-related protein (*ADRP*), prostaglandin reductase 1 (*PTGR1*), nuclear autoantigenic sperm protein (*NASP*), estrogen-related receptor (*ERR*), G protein-coupled receptor family (*GPRs*), *VASA*, and progestin membrane receptor component (*PGRMC1-2*) genes, *etc* [37–42].

### SSR and SNP discovery

**Fig 7:**
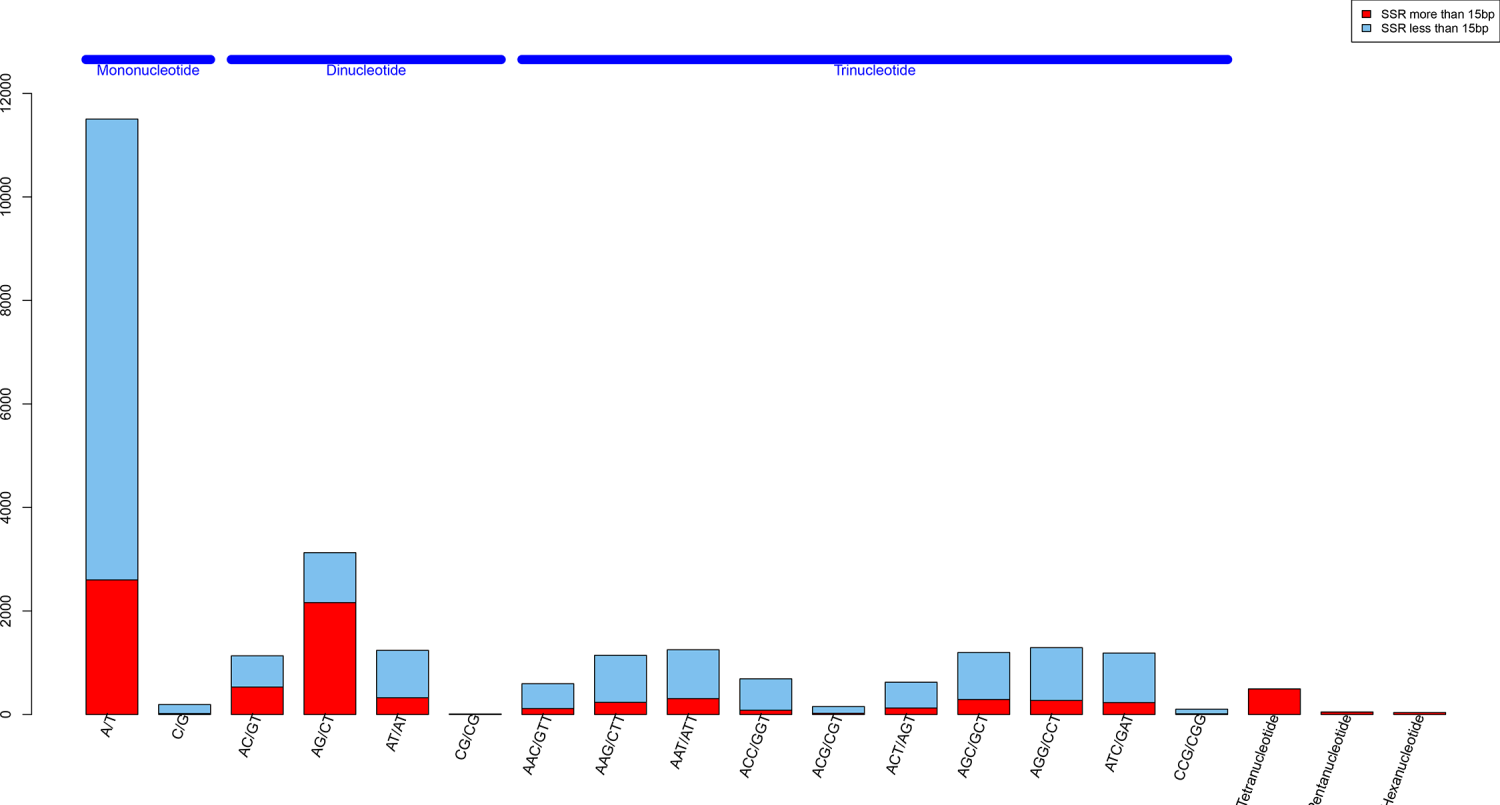
Distribution of simple sequence repeat (SSRs) nucleotide classes among different nucleotide types found in *M*. *nipponense* sequences. X-axis: Distribution of SSR types, Y-axis: The number of different SSR types.

A total of 26,008 SSRs were identified by analysis of the unigenes with MSATCOMMANDER V. 0.8.2 (Fig 7, S6 Table). The largest number of SSR motifs were mononucleotides (11,679), which accounted for 44.91% of all the predicted SSRs, followed by trinucleotides (8,238, 31.67%), dinucleotide (5,511, 21.19%), tetranucleotides (495, 1.90%), pentanucleotide (48, 0.18%) and hexanucleotides (37, 0.14%). In addition, 7,927 SSRs in the whole predicted SSRs were longer than 15bp, which comprised 2,621 mononucleotide SSRs, 3,022 dinucleotide SSRs, 1,704 trinucleotide SSRs, 495 tetranucleotide SSRs, 48 pentanucleotide SSRs and 37 hexanucleotide SSRs.

**Fig 8:**
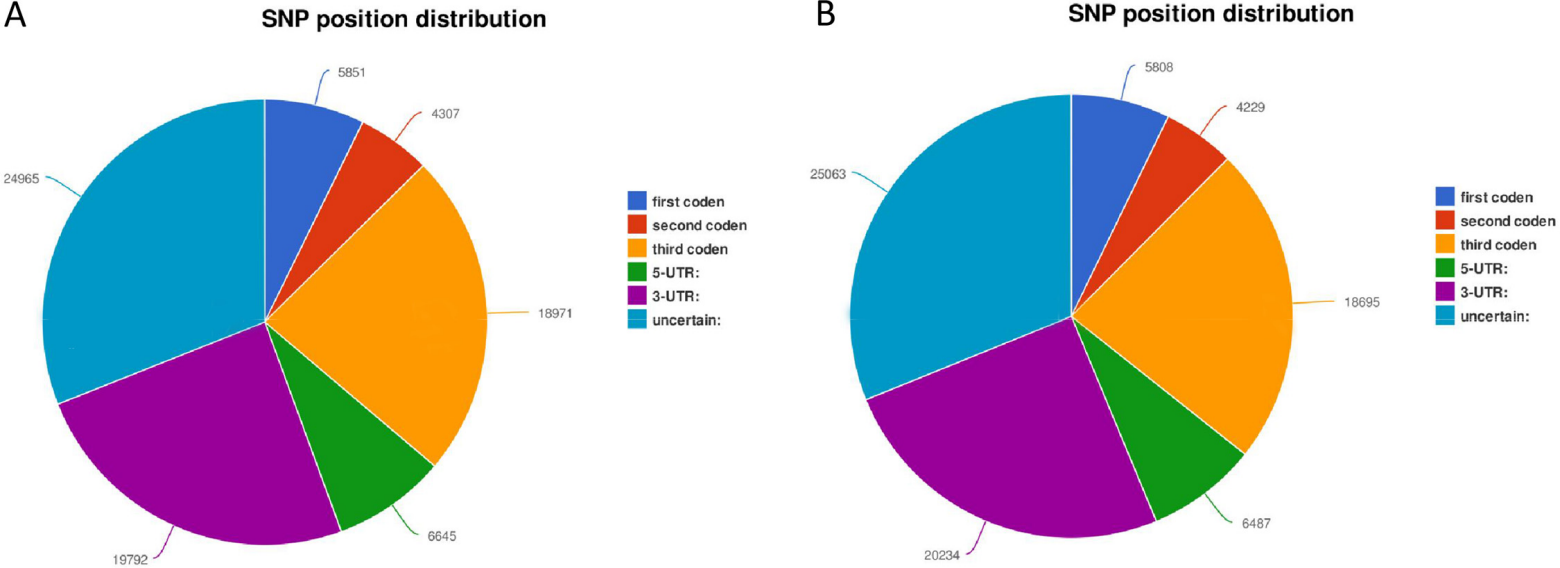
Positions of single nucleotide polymorphisms (SNPs). **A** SNP positions in the transcriptomes of MNOP. **B** SNP position in the transcriptomes of MNON.

Using the Samtools and VarScan v.2.2.7 software, we predicted 80,529 potential SNPs in MNOP and 80,516 potential SNPs in MNON when all the consensus assembly sequences generated from the two transcriptome libraries were employed as reference sequences. The predicted SNPs in MNOP included 48,223 transitions (59.88%) and 32,306 transversions (40.12%), and the predicted SNPs in MNON included 48,051 transitions (59.68%) and 32,465 transversions (40.32%). The most frequent SNPs types in both MNOP and MNON were C/T, A/G and A/T, while C/G was the least common type (Table 3). Of all the SNPs in MNOP, 5,851 (7.27%), 4,307 (5.35%) and 18,971 (23.56%) SNPs were located at the first, the second and the third position of the codon, respectively, 6,645 (8.25%) and 19,792 (24.58%) SNPs were located at 5'UTR and 3'UTR, respectively (Fig 8A). Of all the SNPs in MNON, 5,808 (7.21%), 4,229 (5.25%), 18,695 (23.22%), 6,487 (8.06%) and 20,234 (25.13%) SNPs were located at the first, the second and the third position of the codon, 5'UTR and 3'UTR, respectively (Fig 8B). In addition, we also predicted 29,851 potential SNPs between MNON and MNOP groups (Table 4). These predicted SNPs included 17,570 transitions (58.86%) and 12,281 transversions (41.14%). 2,019 (6.76%), 1,495 (5.01%) and 6,471 (21.68%) SNPs were located at the first, the second and the third position of the codon, respectively, 2,181 (7.31%) and 7,454 (24.97%) SNPs were located at 5'UTR and 3'UTR, respectively.

**Table 3:** Statistics of SNP types in the transcriptomes of MNOP and MNON when all the consensus assembly sequences generated from the two transcriptome libraries were employed as reference sequences.

	MNOP			MNON	
SNP types	Count	Frequence per kb	SNP types	Count	Frequence per kb
Transition	**48,223**		Transition	**48,051**	
C/T	23,967	0.30	C/T	23,897	0.29
A/G	24,256	0.30	A/G	24,154	0.30
Transversion	**32,306**		Transversion	**32,465**	
A/T	11,853	0.14	A/T	11,977	0.15
A/C	7,906	0.09	A/C	7,967	0.09
T/G	8,317	0.10	T/G	8,259	0.10
C/G	4,230	0.05	C/G	4,262	0.05
Total	**80,529**	1.01	Total	**80,516**	1.01

**Table 4:** Statistics of SNP types and positions in the transcriptomes of MNOP when all sequences generated from the MNON transcriptome library were employed as reference sequences.

	SNP types in MNOP		SNP positions in MNOP	
SNP types	Count	Frequence per kb	SNP positions	Count	
Transition	**17,570**		The first position of thecodon	2,019	
C/T	8,735	0.10	The second position of thecodon	1,495	
A/G	8,835	0.11	The third position of thecodon	6,471	
Transversion	**12,281**		5'UTR	2,181	
A/T	4,492	0.05	3'UTR	7,454	
A/C	3,069	0.03	Uncertain	10,231	
T/G	3,005	0.03			
C/G	1,715	0.02			
Total	**29,851**	0.37	Total	**29,851**	

### Identification and validation of differentially expressed genes

**Fig 9:**
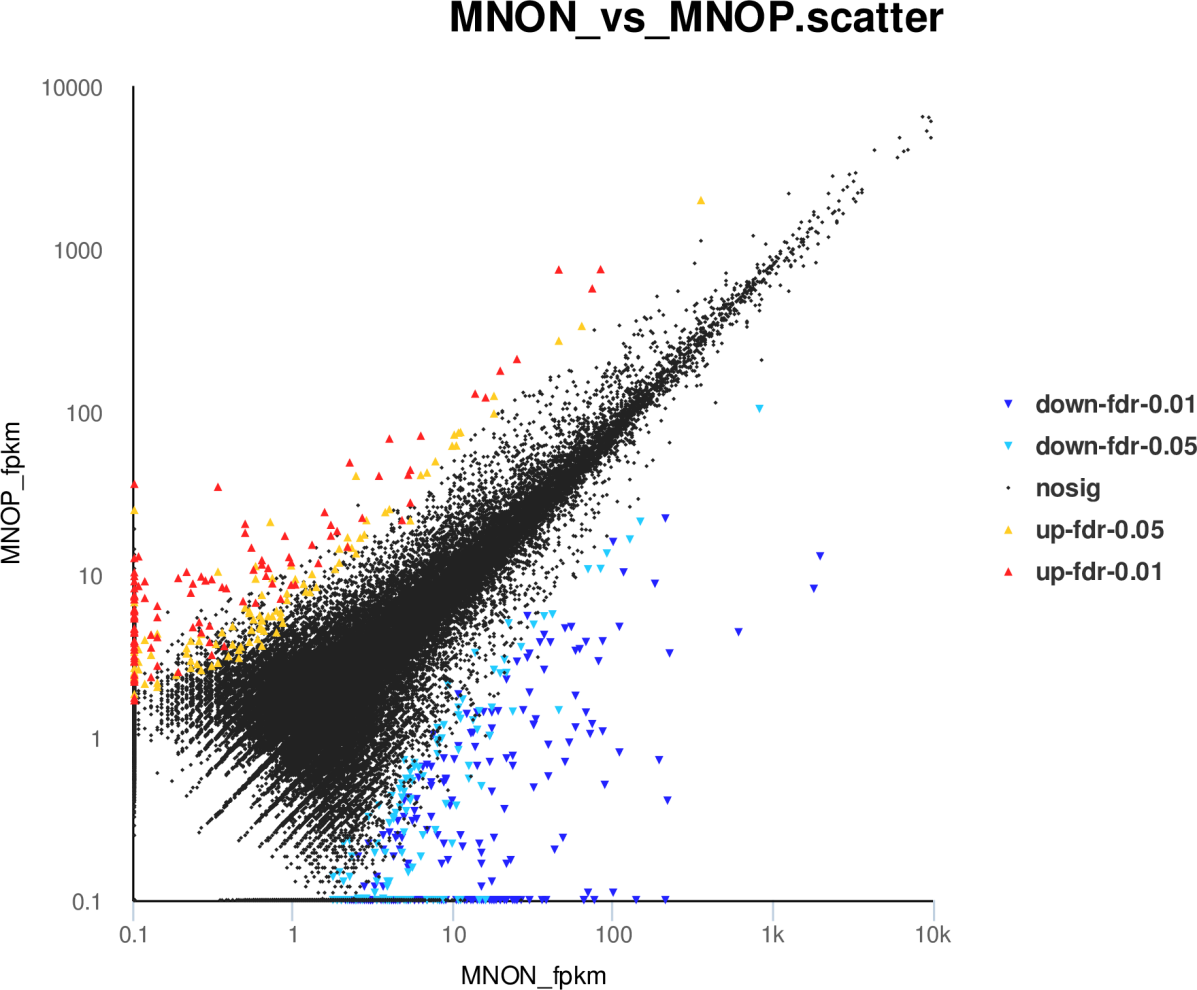
Scatter plot of differentially expressed unigenes (DEGs) between MNOP and MNON. The yellow point (0.01 < fdr < 0.05) and red point (fdr < 0.01) represent significant up-regulated unigenes, the light blue point (0.01 < fdr < 0.05) and dark blue point (fdr < 0.01) represent significant down-regulated unigenes, the black point is no significant difference unigenes.

**Fig 10:**
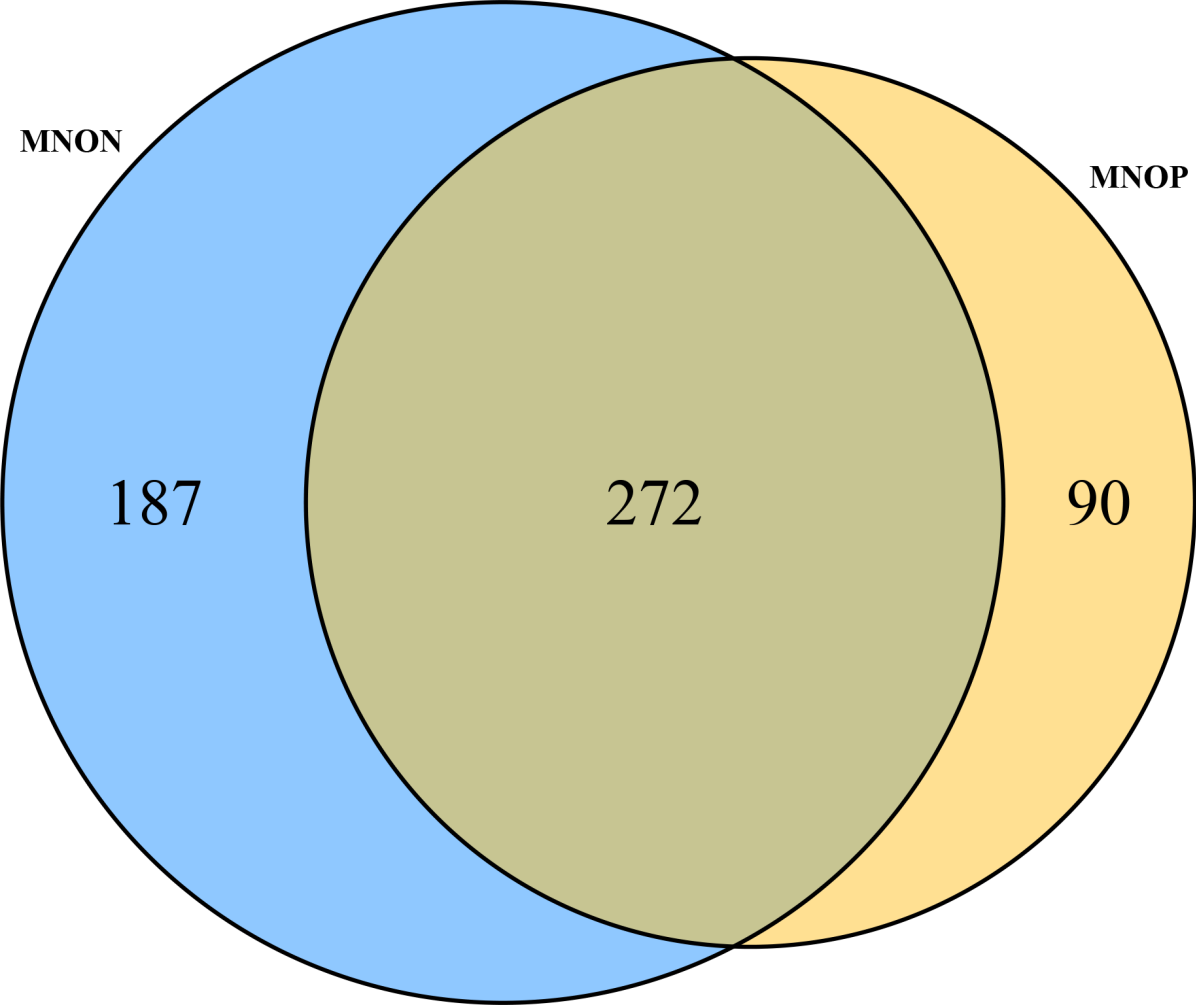
Venn diagram for exclusive DEGs found in MNON and MNOP. The number of the shared DEGs are in the cross area, while the number of the specific DEGs are in the single area.

Through differential expression analysis (FDR ≤ 0.05, logFC ≥ 1) using edgeR, 549 unigenes (0.87% of all unigenes) were identified as DEGs between MNOP and MNON, which comprised 212 up-regulated unigenes (38.62% of all DEGs) and 337 down-regulated unigenes (61.38% of all DEGs) in MNOP compared with MNON. However, 62,787 unigenes (99.13% of all unigenes) had no significant difference between MNOP and MNON (FDR > 0.05) (Fig 9, Table 5, S7 Table). Moreover, 187 MNON-specifically expressed unigenes and 90 MNOP-specifically expressed unigenes were identified from the 549 DEGs, and the remaining 272 DEGs were common unigenes expressed both in the MNOP and MNON (Fig 10). Of 549 DEGs, 186 (33.88%) unigenes were annotated successfully, and the remaining 363 unigenes had nearly no information. Based on the published literature and sequence annotations, we identified nine reproduction-related genes that may be critical for sexual precocity of *M*. *nipponense* from those annotated DEGs (Table 6). They include *vitellogenin* (*VTG*), *cathepsin L* **(***CTSL*), *cystatin-M* (*CST6*), *insulin-like receptor* (*INSR*), *insulin-like growth factor 1 receptor* (*IGF1R*), *cyclooxygenase* (*COX2*), *glutathione peroxidase* (*GPX*), *copper zinc superoxide dismutase* (*SOD1*), and *fatty acid synthase* (*FASN*) genes which involved in ‘ovary development’, ‘ovarian steroidogenesis’, ‘Peroxisome’, ‘Glutathione metabolism’ and ‘Fatty acid biosynthesis’ pathways. *COX2 and SOD1* genes were down-regulated, while *VTG*, *CTSL*, *CST6*, *INSR*, *IGF1R*, *GPX* and *FASN* genes were up-regulated in MNOP compared with MNON.

**Table 5:** The differential expression unigenes (DEGs) between MNOP and MNON.

Items		MNOP versus MNON transcriptome	
	All^a^	Up^b^	Down^c^
Differential expression unigenes (DEGs)	549	212	337
DEGs: fdr < 0.01	316	104	212
DEGs: 0.01 < fdr < 0.05	233	108	125
No Significant difference unigenes: fdr > 0.05	62,787		

^a^ Means summary of unigenes expressed up and down between MNOP and MNON transcriptome

^b^ Indicates number of up-regulated unigenes in MNOP compared with MNON

^c^ Means number of down-regulated unigenes in MNOP compared with MNON

**Table 6:** Nine key reproduction-related DEGs that affect sexual maturity of *M*. *nipponense*.

Pathway	Unigene ID	Gene name	log2FC(MNOP/MNON)	FDR	Regulate
Ovary development	c33992_g1	*Vitellogenin* (*VTG*)	5.45	1.58E-09	Up
	c25097_g1	*Cathepsin L* (*CTSL*)	4.01	2.21E-04	Up
	c31957_g1	*Cystatin-M* (*CST6*)	2.48	3.49 E-02	Up
Ovarian steroidogenesis	c23275_g1	*Insulin-like receptor* (*INSR*)	2.65	3.31 E-02	Up
	c33061_g1	*Insulin-like growth factor 1 receptor* (*IGF1R*)	2.44	3.33 E-02	Up
	c13375_g1	*Cyclooxygenase* (*COX2*)	-3.2	4.72 E-03	Down
Glutathione metabolism	c25682_g1	*Glutathione peroxidase* (*GPX*)	3.00	6.28 E-03	Up
Peroxisome	c8193_g1	*Copper zincsuperoxide dismutase* (*SOD1*)	-2.69	3.31 E-02	Down
Fatty acid biosynthesis	c32305_g1	*Fatty acid synthase* (*FASN*)	2.61	2.13 E-02	Up

Regulate: Up-regulated or down-regulated of genes in MNOP compared with MNON

**Fig 11:**
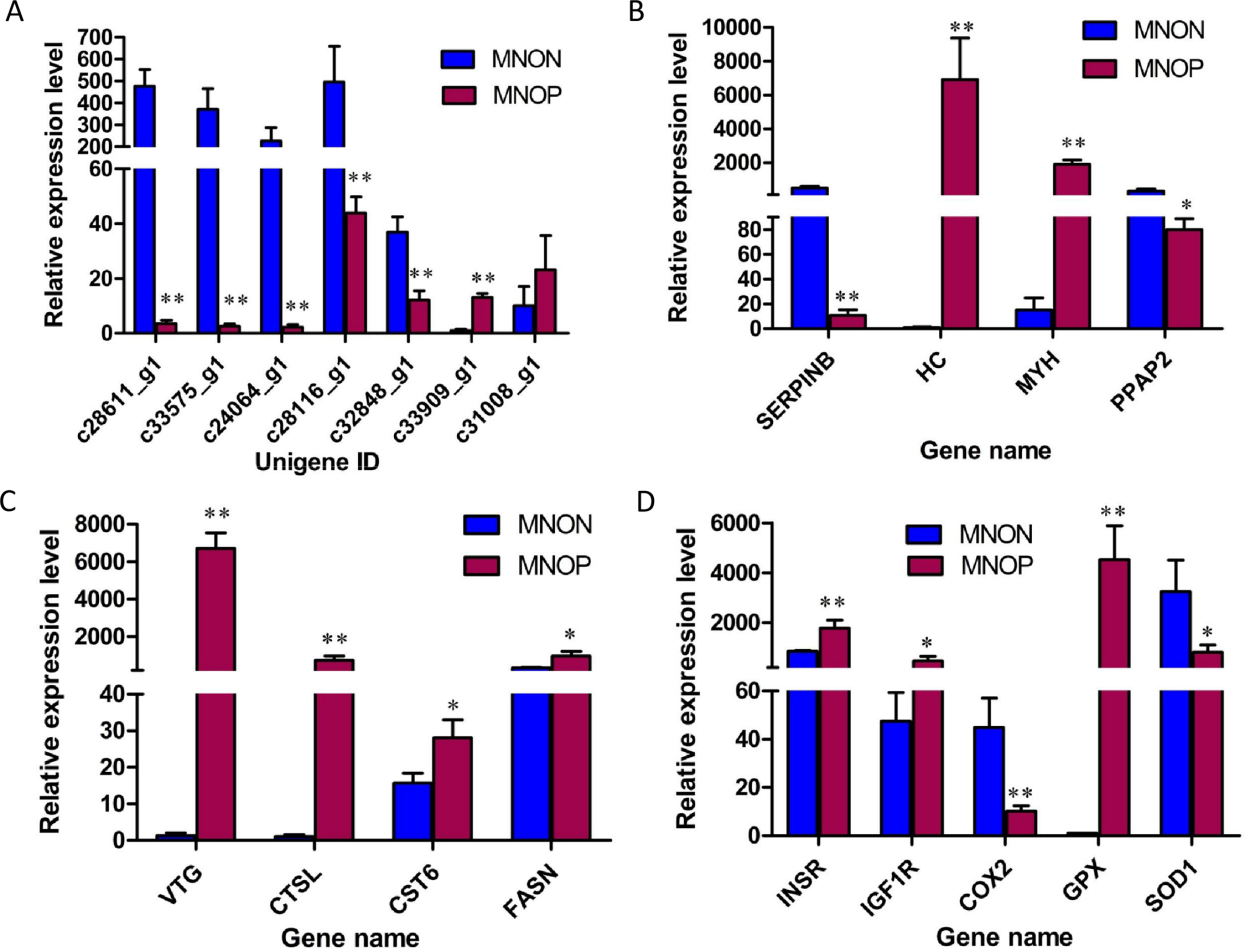
QPCR analysis of twenty selected DEGs. X-axis: The 20 differentially-expressed unigenes or genes, Y-axis: Relative expression level of each unigenes or genes. Beta-actin is as internal control gene. * indicates significantly differential expression (*P* < 0.05), ** indicates most significantly differential expression (*P* < 0.01).

Here, we used Quantitative real-time PCR (QPCR) to confirm the differential expression of 20 DEGs (include those nine genes that may be critical for sexual precocity of *M*. *nipponense*) (S7 Table). As shown in Fig 11, the relative expression levels of five unannotated unigenes (unigene ID: c28611_g1, c33575_g1, c24064_g1, c28116_g1, c32848_g1) and two annotated genes (*SERPINB* and *COX2*) showed significant down-regulation in MNOP compared with MNON, and the relative expression levels of one unannotated unigene (unigene ID: c33909_g1) and eleven annotated genes (*HC*, *MYH*, *PPAP2*, *VTG*, *CTSL*, *CST6*, *FASN*, *INSR*, *IGF1R*, *GPX* and *SOD1*) showed significant up-regulation in MNOP compared with MNON. These QPCR results all agreed with the results of RNAseq-based differential expression analysis (S1 Table, S7 Table). Only one unannotated unigene (unigene ID: c31008_g1) did not show consistent expression between QPCR and the RNAseq-based differential expression analysis, its relative expression levels showed no significant difference between MNON and MNOP.

## Discussion

Little is known about the genes and biological mechanisms controlling sexual precocity in crustaceans. In this study, by profiling the ovary transcriptomes of sexually precocious and normal sexually mature *M*. *nipponense*, we expand the limited amount of sequence data that are available for *M*. *nipponense* in the public EST database and identified differentially expressed genes potentially influencing the sexual precocity of this prawn.

In this study, the RNA sequencing produced total 63,336 unigenes with average length was 893.88 bp from the ovaries of MNOP and MNON. This is considerably higher than the sum of unigenes (1,514) and average length of unigenes (734) obtained from ovary of *M*. *nipponense* using traditional method of expressed sequence tag (EST) reported by Wu et al. (2009). For one thing, the results show that high-throughput RNA-Seq technology has great superiority in large-scale transcriptome studies. For another, this discrepancy probably due to the difference of ovarian development stage of *M*. *nipponense* used in these two studies. Wu et al. (2009) used ovary at previtellogenesis stage as their experimental material, while the ovaries used in this study were at maturation stage. Besides, we used two sequencing samples (ovaries of sexually precocious and normal sexually mature *M*. *nipponense*), giving rise to a huge amount of raw data as compared to the only one sequencing sample (ovary of healthy adult prawn) that was used for transcriptome analysis in study of Wu et al. (2009). To the best of our knowledge, this is the first comprehensive ovary transcriptome study of not only normal sexually mature but also sexually precocious *M*. *nipponense*. Therefore, this transcriptome dataset provides a useful resource for future analyses of genes related to reproduction, gonadal development and sexual precocity in *M*. *nipponense*.

A total of 15,134 (23.89%) unigenes were annotated successfully in the present study, whereas the rest 48,202 (76.11%) unigenes had no annotations which will make a meaningful contribution to the knowledge of *M*. *nipponense* by further characterizing their functions. BLAST results against the Nr database showed that the *M*. *nipponense* sequences had the highest number of matches with the gene sequences from *Z*. *nevadensis*, followed by *D*. *pulex*. *Z*. *nevadensis* was the latest arthropod of the class insecta and *D*. *pulex* was the first arthropod of the class crustacea to have their whole genome sequenced [43,44]. However, only about 11.92% and 7.19% of all the annotated sequences matched *Z*. *nevadensis* and *D*. *pulex* respectively, probably because *Z*. *nevadensis* belongs to pterygota insect, *D*. *pulex* belongs to branchiopoda crustacean, while *M*. *nipponense* belongs to malacostraca crustacean. In addition, the transcriptome research in *L*. *vannamei* was the most extensive of all decapod crustaceans of the suborder Natantia in recent years [45–50], therefore, it is not surprising that *L*. *vannamei* was the only decapod crustaceans of the suborder Natantia in the top twenty species that match to the sequences of *M*. *nipponense*.

The results of GO and KEGG analysis extend our knowledge on the gene function classification and potential involvement of genes in cellular metabolic pathways [51,52]. In this study, the *M*. *nipponense* ovarian sequences fall into GO categories with a roughly similar distribution to that of some other arthropods, such as *Drosophila melanogaster*, *Parhyale hawaiensis*, *D*. *pulex* [53] and *Oncopeltus fasciatus* [54], in which most ovarian sequences were enriched in functional category of “catalytic activity”, suggesting that arthropod ovaries contain a large diversity of enzyme genes involved in various molecular function. As might be expected, many reproduction-related pathways and genes were found in the KEGG analysis, which maybe play an important role in development of oocytes and ovaries in crustaceans. However, further studies are also needed to understand the molecular functions of some putative reproduction-related genes in these pathways in *M*. *nipponense*.

The focus of our project was to mine the differentially expressed genes (DEGs) between MNOP and MNON. In the present study, we identified 549 DEGs which would give clues on the molecular mechanism of sexual precocity determination. In these DEGs, the number of down-regulated unigenes (337) was more than the number of up-regulated unigenes (212), as well as the number of MNON-specifically expressed unigenes (187) was more than the number of MNOP-specifically expressed unigenes (90), indicating that the lack or low expression of quite a few genes in the ovarian development of *M*. *nipponense* is one of the important causes of sexual precocity of this species. Besides, in these DEGs, the number of unannotated sequences (363) was much larger than the annotated ones (186), which made it impossible to exploit all DEGs, some potentially useful genetic information relate to sexual precocity of *M*. *nipponense* may be missed. From these 186 annotated DEGs, we identified nine genes of interest that may be play an important role in sexual precocity of *M*. *nipponense*. They were *VTG*, *CTSL*, *CST6*, *INSR*, *IGF1R*, *COX2*, *GPX*, *SOD1* and *FASN* genes.

*VTG*, *CTSL*, *CST6* are ‘ovary development’- related genes. The expressions of these three genes were significantly up-regulated in MNOP compared with MNON in the present study. VTG is the precursor of vitellin (VN), the major yolk protein that provides nutrition during embryonic development. VTG is synthesized extraovary or intraovary, transported by the hemolymph and finally sequestered into the growing oocytes through vitellogenin receptor (VTGR) mediated endocytosis [55,56]. In oviparous animals, oocyte development and ovarian maturation depend on massive production and accumulation of VTG. In vertebrates, estrogen hormones play a key role in regulating the synthesis of VTG through the enhancement of the transcription of the VTG gene [57,58]. This process is mediated by an E-ER-HSP90-VTG regulation pathway which widely existed in vertebrates [56–61]. During vitellogenesis, estrogen (E) binds to its receptor (ER) form a hormone-receptor complex and then acts on estrogen responsive elements (ERE) located at the upstream of the VTG DNA, which leads to the activation or enhancement of the VTG gene transcription [61]. However, HSP90 binds to estrogen receptor (ER) in the absence of estrogen (E) to increase the activity of estrogen hormone receptor complex to transcribe target genes [59,60,62,63]. In our transcriptomic data, *HSP90* genes were identified. Although an *ER* gene has not been found yet, we identified estrogen related receptor (*ERR*) gene in our transcriptome database (S5 Table). However, *HSP90* and *ERR* genes had no significantly differences between MNOP and MNON. So, whether estrogen (E) caused the significant up-regulation of *VTG* gene expression in MNOP pass through the E-ER-HSP90-VTG regulation pathway need to be confirmed by more studies. After all, the regulatory mechanism of E-ER-HSP90-VTG pathway for VTG synthesis in crustaceans is still controversial [13,37]. Cathepsins are lysosomal cysteine proteases, but cystatins are natural tight-binding reversible inhibitors of cysteine proteases. Based on sequence homology and conserved amino acid motifs, the group of cathepsins can be divided into three subgroups: cathepsin L (CTSL), cathepsin B (CTSB) and cathepsin F (CTSF) [64]. CTSL plays an important role during yolk formation and processing in vertebrates, for example, CTSL can degrade VTG into smaller yolk proteins which are stored in oocyte and will be the primary source of nutrients and energy for the developing embryo [65]. Current findings in *Metapenaeus ensis* [66] and *M*. *nipponense* [67] suggest that the ovary is the unique non-digestive organ showing *CTSL* expression and *CTSL* in the ovary in crustacean is also functionally important in reproductive physiology. On the contrary, as a cysteine protease inhibitor, CST6 plays an important role in regulating and protecting against uncontrolled proteolysis by cathepsins L (CTSL) and V (CTSV) and the asparaginyl endopeptidase legumain (LGMN) [68]. In the present study, the significant up-regulation of *CST6* gene expression in MNOP ovary means that the large amounts of CST6 produced and inhibited the hydrolysis of VTG by CTSL. Although CTSL also produced abundantly because of the significant up-regulation of *CTSL* gene expression, the inhibition of CST6 probably overwhelmed the hydrolysis of CTSL. In sum, the magnificent VTG production due to the significant up-regulation of *VTG* gene expression, together with the inhibition of the hydrolysis of VTG by CST6, led to the accumulation of large amounts of VTG in the ovary, and finally resulted in the sexual precocity of *M*. *nipponense*.

*INSR*, *IGF1R*, *COX2* genes were three DEGs in ‘ovarian steroidogenesis’ pathways (Table 3). Compared with MNON, *INSR* and *IGF1R* gene expressions were significantly up-regulated, while *COX2* gene expression was significantly down-regulated in MNOP. INSR and IGF1R are transmembrane receptors that are activated by hormones called insulin and insulin-like growth factor 1 (IGF-1) respectively. Insulin is a key peptide hormone that regulates growth, metabolism and reproduction in vertebrates [69], However, insulin-like peptides (ILPs), the counterpart of vertebrate insulin, belong to the insulin-like superfamily and were widely found in invertebrate species [70–72]. The insulin-like superfamily includes two major subgroups, ILPs and the insulin-like growth factors (IGFs) [72]. IGF-I is a member of this superfamily, its function overlaps with that of insulin/ILPs [73]. In vertebrates, insulin and IGF-1 banded to the INSR and IGF1R respectively, and then they can stimulate DNA synthesis and promote the proliferation of ovarian theca-interstitial cell through insulin signaling pathway [74]. Insulin signaling pathway is also present in invertebrates, and is closely related to their reproduction. For instance, insulin-like growth factor signaling pathway in *Drosophila* was considered important for the regulation of vitellogenesis, oogenesis, and reproductive diapause [75]. The interaction and crosstalk between ILP signaling networks in *Aedes aegypti* was thought to be relevant to the regulation of yolk protein precursor, juvenile hormone, ecdysteroids, and nutrients [76]. In the present study, the significantly up-regulation of *INSR* and *IGF1R* gene expressions in MNOP means that a great deal of ILPs and IGF-1 generated in the sexually precocious prawn, which promoted vitellogenesis, oogenesis and proliferation of ovarian theca-interstitial cell, leading to the rapid ovarian maturation of *M*. *nipponense*. Cyclooxygenase-2 (COX2) is an inducible enzyme that catalyzes the formation of prostaglandins (PGs) from arachidonic acid (AA). In crustaceans, PGs have important significance in the regulation of female reproductive maturation. In *Procambarus paeninsulanus* [77] and *Oziothelphusa senex senex* [78], there has a positive correlation between the production of PGs (PGE_2_ and PGF_2α_) and the ovarian maturation. Furthermore, the amount of PGs in ovaries of crustaceans varies along with developing ovary stages. For instance, the amounts of PGE_2_ and PGF_2α_ were highest in ovaries at stage I and continued to decrease until stage IV in *Metanephrops japonicus* [79]. In this study, *COX2* gene expression was significantly down-regulated in ovary at stage V of MNOP compared with MNON, suggesting the decrease of PGs in ovary at stage V of sexually precocious *M*. *nipponense*. We believe that the expression of *COX2* gene in immature ovaries (stageⅠ-Ⅵ ovaries) of sexually precocious *M*. *nipponense* was higher than that in mature ovary (stage V ovary) as *M*. *japonicus*, which promoted the synthesis of the PGs in large quantities in immature ovaries, and finally resulted in the rapid ovarian maturation. However, due to the reduced need for PGs in mature ovary, the expression of *COX2* gene decreased sharply in the stage V ovary of sexually precocious *M*. *nipponense*, even significantly lower than that in the stage V ovary of normal sexually mature prawn. But the real expression levels of *COX2* gene in ovaries at different stages of sexually precocious and normal sexually mature *M*. *nipponense* require further investigation.

Aerobic metabolism in animals is associated with the generation of free radicals or reactive oxygen species (ROS) that include the hydroxyl radicals, superoxide anion, hydrogen peroxide, and nitricoxide. ROS have been recently recognized as key signaling molecules involved in reproductive biology including oocyte maturation, fertilization, and embryo development [80–82]. However, because of the double-edged sword property of ROS, excessive ROS can also lead to serious damage to major cellular components including protein, lipid and DNA [83]. To minimize the oxidative damage caused by ROS, cells possess a range of enzymatic scavenger systems including superoxide dismutase (SOD), catalase (CAT) and glutathione peroxidase (GPX). In this study, SOD and GPX belong to ‘peroxisome’ and ‘glutathione metabolism’ pathways, respectively, while they are important antioxidant enzymes in antioxidant system. SOD first catalyzes the dismutation of superoxide radicals to H_2_O_2_, which is further metabolized to H_2_O and O_2_ by CAT and GPX [84]. GPX, a selenoprotein, plays an important role in detoxifying lipids and hydrogen peroxide, with the concomitant oxidation of glutathione [85]. In this study, compared with MNON, *SOD* and *GPX* gene expressions in MNOP were significantly down-regulated and up-regulated respectively, while *CAT* gene expression had no significant difference between these two groups. There must be a precise balance in ROS production and recycling under the regulation of these antioxidant enzymes for avoiding the oxidative damage to cells in ovary of *M*. *nipponense*. The Low expression of *SOD* gene and the over expression of *GPX* gene disrupted this balance and caused overproduction of ROS. The excess ROS promoted the mature of the ovary as signaling molecules, and finally leading to the sexual precocity of *M*. *nipponense*.

It was reported that lipids (including all kinds of fatty acids) are the essential nutrients which stored in the oocytes of female broodstocks to nourish subsequent embryonic development during ovarian maturation of prawn [86]. Moreover, fatty acids also have effects on growth and reproductive success in female prawns through stimulating ovarian maturation and oocyte differentiation [87,88]. For example, C-20 polyunsaturated fatty acids (PUFAs), include arachidonic acid (AA), eicosapentaenoic acid (EPA) and docosahexaenoic acid (DHA), serve as prostanoid precursors and convert into a variety of prostanoids via enzymatic catalysis. One of the more prominent roles of prostanoids in crustaceans is the regulation of female reproductive maturation [89]. Fatty acid synthase (FASN), a key enzyme in the process of the *de novo* fatty acid synthesis in ‘fatty acid biosynthesis’ pathway, catalyzes all of the reaction steps for synthesizing saturated fatty acids (SFA) from acetyl-CoA and malonyl-CoA in an NADPH-dependent manner [90]. Then, various unsaturated fatty acids (USFA) are synthesized from SFA under catalysis of the fatty acid desaturases. In this study, various fatty acids and prostanoids in ovary of MNOP should be richer than those in ovary of MNON because of the up-regulation of *FASN* gene expression in ovary of MNOP compared with MNON, which may be one of the key factors of sexual precocity of *M*. *nipponense*.

Conventional Quantitative real-time PCR was frequently used to confirm the data obtained from high-throughput sequencing. Nineteen out of twenty selected DEGs from RNA-Seq were confirmed by QPCR, which indicates the high reliability of RNA-Seq for detecting DEGs. The differential expressions of above nine key genes that may be related to sexual precocity of *M*. *nipponense* were also verified with QPCR (Fig 11). Although these nine genes are highly related to reproduction of crustaceans, their involvement in sexual precocity of crustaceans has not yet been reported. Our findings in this study firstly revealed the potential relationship between these nine genes and sexual precocity of *M*. *nipponense*. Nevertheless, conclusion on this point cannot be drawn completely until the regulation pathways of these nine genes in sexual precocity of *M*. *nipponense* are fully understood.

SSRs and SNPs are useful molecular markers that have been widely applied into genetic mapping, population genetic analysis and breeding [35]. In the present study, we identified large volumes of SSRs and SNPs which were a lot higher than those in previous reports on *M*. *nipponense* [2,20,21]. This is a powerful supplement to SSR and SNP databases available for *M*. *nipponense*. Moreover, further in-depth research of the potential SNPs between MNON and MNOP groups predicted in this study will also provide certain help for the understanding of the sexually precocious mechanism of this prawn.

## Conclusion

This is the first comprehensive transcriptome dataset for the ovaries of sexually precocious and normal sexually mature *M*. *nipponense* to be reported. A total of 63,336 unigenes were obtained and a number of reproduction-related unigenes and metabolic pathways were identified. Many potential SSRs and SNPs were predicted and can be used for subsequent marker development, genetic linkage and gene localization analysis. Moreover, 549 DEGs and nine key DEGs that are potentially involved in sexual precocity of *M*. *nipponense* were identified for the first time and are worthy of further investigation. In sum, these findings will serve as the important foundation for insight into the regulatory mechanism of sexual precocity of *M*. *nipponense*, and may also provide some references for future molecular research in freshwater prawn.

## Supporting Information

S1 Table Oligonucleotide primers designed for QPCR of twenty candidate DEGs. (DOC)

S2 Table GO category of the unigenes of *M*. *nipponense*. (XLS)

S3 Table The sumary of COG function classification of the unigenes of *M*. *nipponense*. (XLS)

S4 Table Reproduction-related pathways and genes identified from ovarian libraries of *M*. *nipponense*. (DOCX)

S5 Table KEGG annotation of the unigenes of *M*. *nipponense*. (XLS)

S6 Table Statistics of SSRs types in the transcriptomes of *M*. *nipponense*. (XLSX)

S7 Table The annotations of DEGs bewteen MNOP and MNON. (XLS)
