# Supplementary material for: Insights into Sexual Precocity of Female Oriental River Prawn Macrobrachium nipponense through Transcriptome Analysis
Source: PLoS One. 2016 Jun 9;11(6):e0157173. doi: 10.1371/journal.pone.0157173 (PMC4900531; doi:10.1371/journal.pone.0157173)
Supplement: S1 Table — (DOC) [file pone.0157173.s001.doc]

**Table S1 Oligonucleotide primers designed for QPCR of twenty candidate DEGs**

| Unigene ID | Annotation | Regulate | Forward (5′-3′) | Reverse (5′-3′) |
| --- | --- | --- | --- | --- |
| c28611_g1 | ― | down | CATCCTTTGTTGCTGTTTGTCG | ACTGGGGTTACCGCTGCTCT |
| c33575_g1 | ― | down | GCTTCAAAGCCATCCTCGG | CAACATTGTCACCATCCCTCC |
| c24064_g1 | ― | down | GGTCGTTGGAGCGTTTCTTT | TTGCATCTCAGCCCTGTTATTC |
| c28116_g1 | ― | down | CTTGAGTGCGTGAGCTTTTTG | TGTCGGCATCGGCTTTGT |
| c32848_g1 | ― | down | TAGCAAAAGTGGAAGCCAAAAG | ATGGAGGGGCACCGAAA |
| c33909_g1 | ― | up | AAATCCACTCACCGTAAGTCTAACA | GCTCACAAAGCACCTTCATCA |
| c31008_g1 | ― | up | AATGAGTCACAGCCCAGCG | TTACTACGAGAAAGAGAACACCACC |
| c33813_g2 | Serine proteinase inhibitor (SERPINB) | down | CGTCTTGTTGGTTGGGGTAA | CCAGAGGCCAGCAATCTTTT |
| c33027_g1 | Hemocyanin (HC) | up | GTGAATGACAGCAGTGTAAAGGG | AACGCCACAGGGAAGAAGC |
| c33118_g3 | Myosin heavy chain 1 (MYH) | up | CTGGTGGCAAGGGTGGAAA | CAACGGATGAAGTGAGGCG |
| c29346_g1 | Lipid phosphate phosphohydrolase 2 (PPAP2) | up | CCTCCTCATCGGTTTCGTG | GGGGGTTTGTGCAATCCTTC |
| c33992_g1 | Vitellogenin (VTG) | up | GAGCCCATTTCAATGACCCA | CACTCCCACAACCAATCAAGAC |
| c31797_g1 | Cathepsin L precursor (CTSL) | up | GCCCTTTTACACGCCGTT | CCTCTTCTCCGTAGCCCACT |
| c31957_g1 | Cystatin(CST6) | up | GCCACACTACCCAACACAACC | TCGGCAACAACAATCCTTCA |
| c32305_g1 | Fatty acid synthase (FASN) | up | CAAAACCAGCAGATACAAAGGATG | GGCAGTGAGGACCAAGAAAAAC |
| c23275_g1 | Insulin-like receptor (INSR) | up | GGCACCTTCTTCTTCCGCT | TCACATTGACTGTTCCCACCTC |
| c33061_g1 | insulin-like growth factor 1 receptor (IGF1R) | up | GCAGGGAGTGAGGAATGGAA | TGAGAGCACAGAGGACAACGA |
| c13375_g1 | Cyclooxygenase (COX2) | down | GGAAGCTCTTTGAATCCTTTGTC | CGTGTATTTGTCCCGTGGC |
| c25682_g1 | Glutathione peroxidase (GPX) | up | AGGGAACAAAATCAGCCGC | AGCAAAGATACACCCACCAAGA |
| c8193_g1 | Copper zinc superoxide dismutase (SOD1) | up | CCTGATTGGAGCCTTGTGC | CTGGAATGCCTGTAATGTTGGT |
| *β*-actin | ― |  | AATGTGTGACGACGAAGTAG | GCCTCATCACCGACATAA |

# Regulate: Up-regulated or down-regulated of unigenes in MNOP compared with MNON
