# Supplementary material for: Insights into Sexual Precocity of Female Oriental River Prawn Macrobrachium nipponense through Transcriptome Analysis
Source: PLoS One. 2016 Jun 9;11(6):e0157173. doi: 10.1371/journal.pone.0157173 (PMC4900531; doi:10.1371/journal.pone.0157173)
Supplement: S4 Table — (DOCX) [file pone.0157173.s004.docx]

**Table S4** Reproduction-related pathways and genes identified from ovarian libraries of *M. nipponense*

| Pathways | Gen name |
| --- | --- |
| Ovarian steroidogenesis | *PTGS2(COX2), ADCY1, ADCY2, ADCY3, ADCY5, ADCY6, ADCY8, ADCY9, ALOX5, LDLR, PKA, INSR, GNAS, IGF1R, CYP2J, PLA2G4(CPLA2)* |
| Progesterone-mediated oocyte maturation | *HSP90A,* *ORB, ADCY1, ADCY2, ADCY3, ADCY5, ADCY6, ADCY8, ADCY9, PKA, CDK1(CDC2), CDK2, PKMYT, APC1, APC2, APC3(CDC27), APC4, APC5, APC6(CDC16), APC7, APC8(CDC23), APC10(DOC1), PIK3C, PIK3R, MOS, GNAI, SPDY(RINGO), MAP2K1 (MEK1), CCNB, BUB1, JNK, PIK3C, KRAS(KRAS2), P38, MAD1L, MAD2L2, MAD2, CDH1, IGF1R, PLK1, PDE3, MAPK1_3, RPS6KA(RSK2), CCNA* |
| Wnt signaling pathway | *LRP5_6, CSNK1A, CSNK1E, CSNK2A, CSNK2B, VANGL, FZD1_7, FZD4, FZD9_10, SMAD4, EP300(CREBBP, KAT3), APC, GSK3B, TBL1, PLCB, PPP3C(CAN), PPP3R(CNB), SKP1(CBF3D), SIAH1, PKA, SMAD4, CCND1, BAMBI, CUL1(CDC53), PPN, CTNNB1, WNT3, WNT4, WNT5, WNT10, WNT16, FBXW1_11(BTRC, beta-TRCP), CACYBP(SIP), PSEN1(PS1), RAC1, DVL, RHOA, MYC, CTBP, CAMK2, MAP3K7(TAK1), AXIN1, SFRP5,* *JNK,* *RUVBL1(RVB1, INO80H),* *GPC4,* *ROCK2,* *NLK,* *CPKC,* *TP53( P53),* *LRP5_6,* *WIF1, CCND2, GROUCHO, JUN, PRICKLE,* *RBX1(ROC1),* *SIAH1* |
| MAPK signaling pathway | *TEAD, GLO1, MAPK7,* *RHOA, CDC42, PAK1,* *P38, CPKC,* *TPR,* *WSC* |
| Oocyte meiosis | *ORB, ADCY1, ADCY2, ADCY3, ADCY5, ADCY6, ADCY8, ADCY9, CALM, PPP1C, PPP2C, PPP3C(CAN), PPP2R1, PPP2R5, APC1, APC2, APC3(CDC27), APC4, APC5, APC6(CDC16), APC7, APC8(CDC23), APC10(DOC1),* *CALM, SKP1(CBF3D), SMC1, PKA, CUL1(CDC53), FBXW1_11(BTRC, beta-TRCP), MOS, SMC3(CSPG6), ESP1, SPDY(RINGO), YWHAE, CCNE, MAP2K1(MEK1), AURKA, CAMK2, BUB1, PKMYT, CDK1( CDC2), CDK2, CDC20, PPP1C, PPP2C, PPP2R5, PPP3R(CNB), SLK, AURKA, IGF1R, PLK1, ITPR1, YWHAB_Q_Z, MAD2, MAPK1_3, MAD2L2, RBX1(ROC1), RPS6KA(RSK2)* |
| GnRH signaling pathway | *MAP2K1(MEK1), MAP2K4(MKK4), MAP2K6(MKK6), MAP2K7(MKK7), MAP3K1(MEKK1), MAP3K4(MEKK4), MAPK1_3, MAPK7, ADCY1, ADCY2, ADCY3, ADCY5, ADCY6, ADCY8, ADCY9, CALM, PLCB, PKA, ATF4(CREB2), GNAQ, PLD1_2, GRB2, SRC, GNAS,* *PLA2G4(CPLA2), MMP14, CDC42, CAMK2,* *PLD1_2, GNA11, EGFR(ERBB1), SOS, JNK, KRAS(KRAS2), P38, ITPR1, GNRHR, JUN* |
| Estrogen signaling pathway | *HSP90A, HSP90B, CREB3, HSPA1_8, CALM, ADCY1,* *ADCY2*, *ADCY3, ADCY5, ADCY6, ADCY8, ADCY9, PLCB, PKA, PRKCD, ATF2(CREBP1), ATF4(CREB2), GNAQ, PIK3C, PIK3R, GRB2, SRC, GNAS, GNAI, MAP2K1(MEK1), GNAO(G-ALPHA-O), EGFR(ERBB1), SOS, CREB3, SHC1, FKBP4_5, KRAS(KRAS2),* *ITPR1, JUN, MAPK1_3* |
| p53 signaling pathway | *CASP8, RRM2, SIAH1, CCNB, CCND1, CCND2, SESN, MDM2, EI24, TP73, CHK1, CHK2, CCNE, DDB2, CCNG2, RFWD2(COP1), CDK1(CDC2), CDK2, CDK4, CDK6, ATR, ATM(TEL1), TSC2, PPM1D(WIP1), TP53(P53), CYC, SIAH1, PTEN* |
| Steroid hormone biosynthesis | *UGT, SULT1E1(STE), CYP3A, CYP2B, CYP2D, HSD17B12(KAR, IFA38), SRD5A1, SRD5A3* |
| Fatty acid biosynthesis | *ACSBG, fabD, fabF, FASN, ACAC, accD* |
